# Focus on advanced materials for energy harvesting: prospects and approaches of energy harvesting technologies

**DOI:** 10.1080/14686996.2018.1491165

**Published:** 2018-08-02

**Authors:** Hiroyuki Akinaga, Hiroyuki Fujita, Masaki Mizuguchi, Takao Mori

**Affiliations:** a Nanoelectronics Research Institute, National Institute of Advanced Industrial Science and Technology, Tsukuba, Japan; b Institute of Industrial Science, The University of Tokyo, Tokyo, Japan; c Institute for Materials Research, Tohoku University, Sendai, Japan; d International Center for Materials Nanoarchitectonics (WPI-MANA), National Institute for Materials Science, Tsukuba, Japan

**Figure 1.**
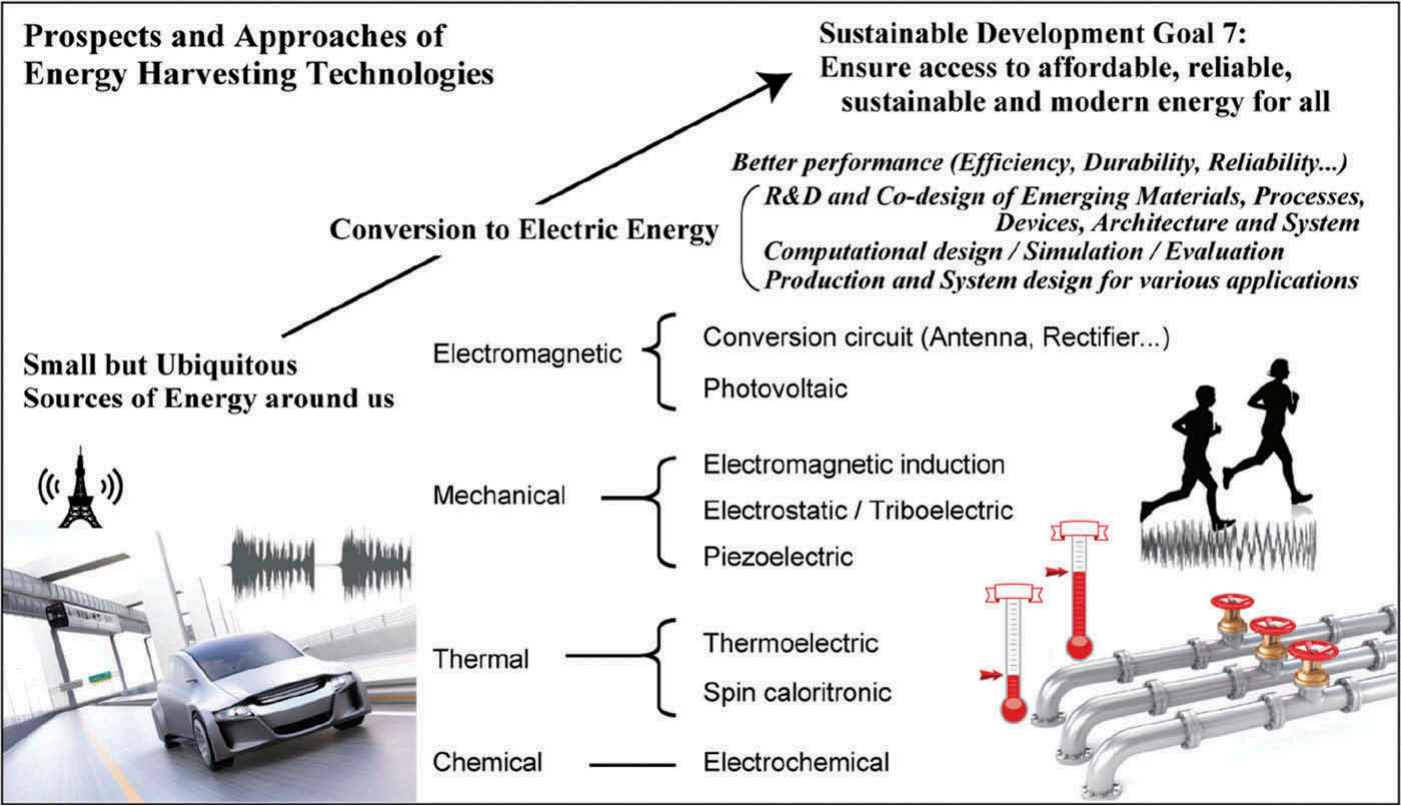
Prospects and approaches of energy harvesting technologies. Energy conversion processes from various energy sources and approaches to improve the harvesting performance are shown.

Energy supplies for wireless communications and sensors will be essential for the future Internet of Things (IoT) society. Environmentally friendly generation of electric energy will be a major concern in a resilient society [1,2]. We have to extend the concept of the power source, from batteries to using ubiquitous energy as power sources, that is, energy harvesting. The interdisciplinary research on energy harvesting has been focused on technologies for the conversion of various ubiquitous energy sources, such as heat, light, vibration, or electromagnetic fields, into the electric energy (see Figure 1).

Materials will play an important role in improving the conversion efficiency. For example, the thermoelectric conversion requires materials with high electrical conductivity and Seebeck coefficient and low thermal conductivity, which is hard to achieve simultaneously. Nanostructuring of the targeted material can break the antagonistic relationship between the electrical and thermal conductivities and yield optimal properties. Novel methods, such as band engineering and/or utilizing magnetism, for example, are being implemented to overcome the conventional trade-off between the Seebeck coefficient and electrical conductivity.

In particular, utilizing spin in thermoelectric and other energy conversion systems is a promising and challenging idea. This topic is expanding as a newly established research field, ‘spin caloritronic energy conversion technology’. Its challenges include the development of proper figures of merit and of the quantitative evaluation methods for novel energy harvesting materials and nanostructures.

In vibration-driven electret energy harvesters, an appropriate electret is required for increasing the surface electrical potential. The high-density charge of the electret may affect static adhesion and friction forces, thereby hindering the expected motion of the device. A system design combining allaspects of the fabrication process, electromechanical structures, and a power conversion circuit must be conducted to increase the efficiency of the power generation.

This focus issue of *Science and Technology of Advanced Materials* will hopefully stimulate the research and development of energy harvesting as a robust tool for the sustainable development of our society. We sincerely hope this focus issue will foster collaborations among a wide range of scientific communities and spur the future advancement of energy harvesting.
